# Effectiveness of aortic balloon occlusion in reducing blood loss during cesarean section in placenta accreta spectrum disorders: a study protocol for a randomized controlled trial

**DOI:** 10.3389/fmed.2025.1535258

**Published:** 2025-02-24

**Authors:** Gauri Bapayeva, Gulzhanat Aimagambetova, Nazira Kadroldinova, Viktor Zemlyanskiy, Kuat Kassymbek, Milan Terzic

**Affiliations:** ^1^Clinical Academic Department of Women’s Health, CF “University Medical Center”, Astana, Kazakhstan; ^2^Department of Surgery, School of Medicine, Nazarbayev University, Astana, Kazakhstan; ^3^Clinical Academic Department of Radiology and Nuclear Medicine, CF “University Medical Center”, Astana, Kazakhstan

**Keywords:** obstetric hemorrhage, abnormally invasive placenta, endovascular balloon occlusion, cesarean section, REBOA, clinical outcome, cesarean hysterectomy, placenta accreta spectrum

## Abstract

**Introduction:**

Obstetric hemorrhage is one of the leading causes of maternal mortality and morbidity worldwide. One of the major risk factors of obstetric hemorrhage include placenta previa and placenta accreta spectrum (PAS) disorders. The frequency of PAS disorders is increasing worldwide and is accompanied by massive intraoperative bleeding with hemorrhagic shock and increasing rates of cesarean hysterectomy. To decrease the risks of bleeding, various approaches to endovascular balloon occlusion have been tested during the past decade.

**Objectives:**

This study aimed to evaluate the effectiveness of resuscitative endovascular balloon occlusion of the aorta (REBOA) in reducing blood loss and preserving the reproductive organs during cesarean section. Study design: This will be a prospective randomized controlled trial involving 144 patients in a tertiary care obstetric center in Kazakhstan. The study population will consist of pregnant women who will be admitted for cesarean section due to placenta previa complicated by PAS disorders. The study subjects will be randomly divided into intervention and control groups.

**Results:**

The results will be analyzed through the measurement of primary (blood loss during cesarean section) and secondary outcomes [occurrence of hysterectomy during cesarean section, blood transfusion volume, duration of surgery, balloon application time, stay in intensive care unit (ICU), neonatal outcomes, complications, and total days of postsurgical hospital stay].

**Conclusion:**

The use of REBOA is expected to minimize intraoperative blood loss during cesarean section, decrease the need for transfusion of blood components, reduce the time of surgical intervention, decrease the rate of maternal complications, and reduce the rate of cesarean hysterectomy.

## 1 Introduction

Obstetric hemorrhage is a global burden due to its serious impact on maternal morbidity and mortality through mechanisms such as tissue hypoperfusion, disseminated intravascular coagulation (DIC), intensive care unit admission, and multiple organ/system failure (1). One of the major risk factors for obstetric hemorrhage is placenta previa and morbidly adherent placenta (2–5). Moreover, the incidence of placenta previa and abnormally invasive placenta has been growing during recent decades due to increased rates of cesarean delivery worldwide (3, 6).

Several terms are used in the literature to define abnormally invasive placenta, including abnormally adherent placenta, invasive placenta, and placenta accreta spectrum disorders (2, 3). This study will use the placenta accreta spectrum (PAS) disorders classification proposed by FIGO (2, 7). The classification of placental invasion comprises three grades: FIGO 1 – placenta accreta, FIGO 2 – placenta increta, and FIGO 3 – placenta percreta (2, 7). There is a high risk of massive intraoperative hemorrhage during cesarean section in case of PAS disorders leading to cesarean hysterectomy in most of the cases (3). Therefore, the main goal in the management of pathologically adherent placenta is to reduce intraoperative bleeding and perform organ-sparing surgery.

In recent decades, various types of minimally invasive endovascular interventions have been studied to reduce intraoperative blood loss while preserving fertility in women with PAS disorders (8–13). These interventions include uterine artery embolization (UAE) and placement of balloon catheters in the internal iliac arteries and in the aorta (9, 10, 14).

Resuscitative endovascular balloon occlusion of aorta (REBOA) is gaining popularity for abdominal bleeding emergencies and has also been used in obstetric practice since 1995 (15). REBOA is a minimally invasive procedure in which a balloon catheter is inserted into the infrarenal part of the aorta (Zone III; proximal and distal) to control bleeding. In most of the previously reported studies, REBOA is found to be an effective method for reducing intraoperative blood loss and blood transfusion volume at cesarean section in cases of PAS (16–20). These studies concluded that the use of REBOA is associated with a decrease in intraoperative blood loss, a reduction in the need for transfusion of donor blood components, and surgical time. It is also favorable for the conservation of reproductive organ(s) and earlier transfer to the postpartum department/discharge from the hospital.

The advantage of REBOA compared to other endovascular procedures is that the procedure can be performed in a standard operating room, eliminating the need for multiple inconvenient transfers of patients between the interventional radiology department and the operating room or the use of hybrid operating rooms, thereby optimizing resources. Balloon placement is confirmed using an ultrasound device, reducing the risk of potential radiation exposure to the patient, fetus, and personnel (21, 22). Access to the vessel with REBOA is made through a single femoral access compared to the bilateral access required for the occlusion of the internal iliac and uterine arteries, which significantly reduces operating time.

The Republic of Kazakhstan is a middle-income Central Asian country with a population of 20 million with approximately 400,000 annual deliveries (23). Kazakhstan has a growing birth rate over the past decade (24), which increases the demand for high-quality maternity care. Therefore, the Kazakhstani government prioritized maternity healthcare and childcare, increasing the budget to support the healthcare system (23). Currently, the management of PAS cases is guided by the Kazakhstani national protocol on “Pathology of the Placenta” (25). According to this document, cesarean section is recommended at 36–37 weeks of gestation for cases of PAS disorders (25). In the event of intraoperative massive bleeding, the guideline suggests the administration of uterotonic drugs, application of additional hemostatic sutures, and ligation of the arteries supplying the uterus. In cases of PAS, the guideline suggests the application of UAE to minimize surgical bleeding (25). However, if the above methods are ineffective and bleeding continues, further management includes a hysterectomy. Rates of cesarean hysterectomy in Kazakhstan are comparable with other middle-income countries, with cesarean hysterectomy due to placental pathology (placental abruption, placenta previa, and PAS) reported as the second most common indication (26). The national guideline on placental pathologies management does not regulate the use of endovascular interventions in the management of PAS disorders.

Considering that no experience in the use of endovascular interventions for PAS disorders management exists in the Kazakhstani obstetrics care setting, this project will evaluate the effectiveness of REBOA in reducing blood loss and preserving the reproductive organ during cesarean section. In addition to assessing the effectiveness of endovascular treatment, this study will identify the risk factors for thromboembolic complications associated with massive bleeding, which is defined as blood loss of more than 1,500 mL. Identification and stratification of risk groups for thromboembolic complications will allow the selection of patients for minimally invasive interventions in obstetric practice in the future.

The hypothesis is that REBOA will minimize intraoperative blood loss during cesarean section, decrease the need for transfusion of blood components, reduce the rate of hysterectomy need, and decrease the time of surgical intervention and the length of patient stay in the hospital in patients with PAS disorders.

## 2 Materials and methods

### 2.1 Study design and study environment

This will be a prospective, randomized controlled trial (RCT) that will investigate the superiority of REBOA in combination with cesarean section compared to the traditional cesarean section for pregnant women with PAS disorders. The study was registered in the Clinical.Trials.gov database (registration ID NCT06721182) and will take place in the National Research Center for Mother and Childcare (NRCMC), University Medical Center (UMC), Astana, Kazakhstan. The NRCMC is a tertiary care leading specialized institution in the field of obstetrics and gynecology in Kazakhstan. The hospital has sufficient experience in the field of scientific research and a powerful technical and personnel base.

### 2.2 Study subjects

The study population will consist of pregnant women who will be admitted to the NRCMC of UMC for cesarean section due to placenta previa complicated by PAS disorders. The study subjects will be divided into cases and controls. The following inclusion criteria will be applied for the study: (1) singleton pregnancy at 34 weeks of gestation or more, (2) age 18 to 45 years, (3) confirmed diagnosis of placenta accreta spectrum, (4) indication for elective cesarean section, and (5) consent for aortic balloon occlusion during cesarean section.

The following exclusion criteria will be followed for both, case and control groups: (1) pregnant patients at the gestational age < 34 weeks of gestation, (2) multiple pregnancies, (3) need for emergency cesarean section, (4) blood coagulation disorders (known coagulopathy, women with abnormal baseline activated partial thromboplastin time (aPTT), prothrombin time (PT), and international normalization ratio (INR) before surgery). In addition, patients on antiplatelet and anticoagulant drugs with a known history of thrombotic events will also be excluded.

### 2.3 Recruitment process

The study participant recruitment will be performed from December 2024 to December 2025. Subjects who meet the eligibility criteria will be randomly assigned to either the intervention/case group (cesarean section + REBOA) or to the traditional treatment/control group (cesarean section). To ensure the control group patients’ safety, intraoperative bleeding will be managed according to the national guidelines for the pathology of the placenta (25) and postpartum bleeding management.

**Figure 1:**
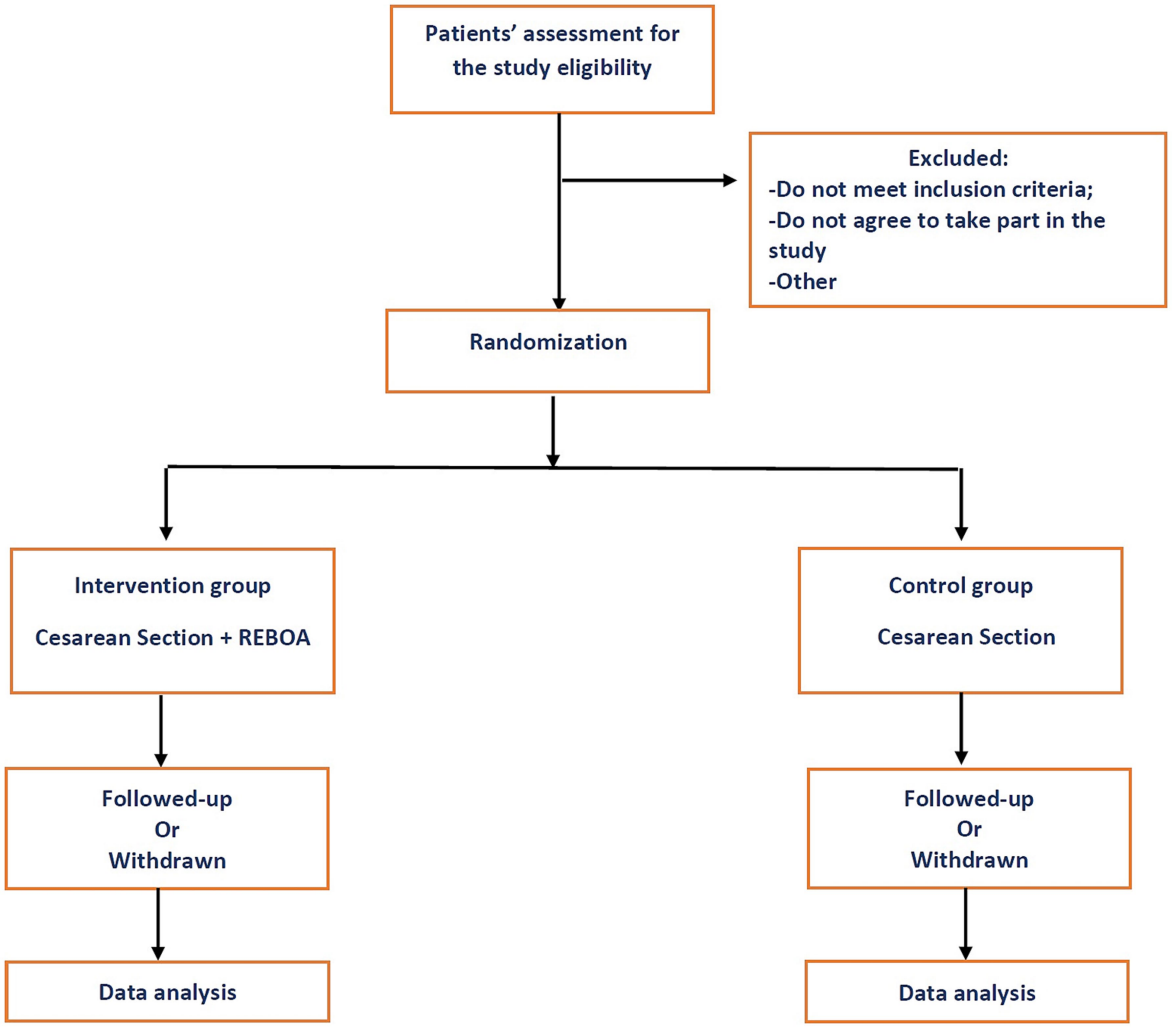
Schematic diagram of the study.

A simple randomization method will be applied (Figure 1). Randomization will be based on a “single sequence of random assignments known as simple randomization” (27). This technique maintains complete randomness of the assignment of a person to a particular group. The randomization procedure includes using a shuffled deck of cards (odd - intervention group, even – control group) (27). The randomization will be performed after patient recruitment, once the study aims and expected outcomes are explained to the patient and informed consent is obtained, by the responsible project team member.

### 2.4 Sample size calculation

To estimate the required sample size, effect sizes for three major outcomes (mean intraoperative blood loss, blood transfusion volume, and hysterectomy rates) were derived from previous studies examining the impact of REBOA on reducing blood loss during cesarean sections in patients with placenta accreta spectrum disorders. Due to substantial heterogeneity in the effect sizes across studies (*I^2^* for mean blood loss difference = 94.89% and *I^2^* for mean transfusion volume = 86.95%), random-effects meta-analyses were conducted on individual study-level blood loss and transfusion volumes extracted from two previously published meta-analyses (28, 29). For the hysterectomy outcome, a fixed-effect meta-analysis was applied due to lower heterogeneity (Q = 19.90, *I^2^* = 29.64%).

The meta-analysis of mean blood loss differences between REBOA and control groups, based on previous studies (28, 29), revealed a random-effects pooled estimate of −1331.46 mL (95% CI: −1643.21 to −1019.71) and a pronounced effect size (Cohen’s *d* = − 2.06). Using the pooled mean difference ( $\Delta_{\mathrm{pooled}}$ ) and variance adjusted for between-study heterogeneity ( $SE^{2}+\tau^{2}$ ), the required sample size was calculated as follows:


$$\begin{array}{l} n=\frac{2\times {\left(Z_{\alpha /2}+Z_{\beta }\right)}^{2}\times \left(SE^{2}+\tau^{2}\right)}{\Delta_{\mathrm{pooled}}^{2}}\\ =\frac{2\times {\left(1.96+0.84\right)}^{2}\times \left(159.06^{2}+292106.16^{2}\right)}{-1331.46^{2}}\\ =2.81\approx 3\;\mathrm{patients}\;\mathrm{per}\;\mathrm{group} \end{array}$$

Similarly, a meta-analysis of blood transfusion volume differences, based on 13 studies, yielded a pooled estimate of −975.10 mL (95% CI: −1224.25 to −725.95) with a similar effect size (Cohen’s *d* = − 2.08), resulting in a required sample size estimate of 2.99 (≈ 3 patients per group). These small sample size estimates reflect the large effect sizes observed, where the pooled mean differences are substantial relative to the pooled standard deviation, yielding a high signal-to-noise ratio. This robustness persists even after accounting for high heterogeneity in continuous outcomes.

For hysterectomy rates, fixed-effect meta-analysis across 15 studies revealed a pooled odds ratio (OR) of 0.31 (95% CI: 0.21 to 0.46) with a significant overall effect (Z = −5.84, *p* < 0.001).

To calculate the required sample size for this outcome, probabilities of hysterectomy were derived from pooled OR ( ${\mathrm{OR}}_{\mathrm{pooled}}$ = 0.31) of meta-analysis using the following formula


$$p_{2}=\frac{p_{1}\cdot {\mathrm{OR}}_{\mathrm{pooled}}}{1-p_{1}+\left(p_{1}\cdot {\mathrm{OR}}_{\mathrm{pooled}}\right)}$$

Subsequently, the sample size was estimated based on calculated probabilities as follows:


$$\begin{array}{l} n=\frac{{\left(Z_{\alpha /2}+Z_{\beta }\right)}^{2}\times \left[p_{1}\left(1-p_{1}\right)+p_{2}\left(1-p_{2}\right)\right]}{{\left(p_{1}-p_{2}\right)}^{2}}\\ \approx 72\;\mathrm{patients}\;\mathrm{per}\;\mathrm{group} \end{array}$$

Using a sample size greater than the largest estimated requirement (n ≈ 72 per group) among major outcomes will ensure sufficient power for evaluating the impact of REBOA in minimizing blood loss during cesarean sections in patients with placenta accreta spectrum disorders, adequately accounting for the detection of previously reported effect sizes with a significance level (*α*) of 0.05 and a statistical power (1−*β*) of 80%. Thus, the calculated sample size was 72 patients per group (overall 144 patients).

### 2.5 Diagnostic methods

All patients will undergo clinical and anamnestic data collection procedures (patient interview for identification of chief complaints, past medical history, and performing external obstetric examination) (Table 1).

**Table 1:** Timeline of procedures within the project.

	Procedures	Before cesarean section (preoperative)	During cesarean section (intraoperative)	After cesarean section (postoperative)
1	Clinical and anamnestic methods	+	−	−
2	Laboratory tests	+	+	+
3	US/MRI diagnostics	+	−	−
4	Pathohistological examination	−	−	+

Laboratory tests (common blood test, biochemical blood test, coagulation tests, and thromboelastography) will be performed following the general examination. Thromboelastography (TEG) will be carried out at the transfusiology department of UMC using the TEG5000 Haemonetics 2017 thromboelastograph. A thromboelastograph is a device used to assess the mechanical strength of clot formation using the interaction between coagulation factors and platelets, which cannot be assessed in other laboratory methods such as coagulation tests (30, 31). A blood test for thromboelastography will be taken at the following time points (1) the preoperative period (24 h before surgery); (2) 10 min after fetal extraction; (3) immediately after closing the skin wound; and (4) 24 h after surgery. The key TEG parameters include reaction time (R), clot formation time (K), maximum amplitude (MA), alpha angle (*α*), percent lysis 30 min after MA (Ly30), and coagulation index (CI; a calculated index of total coagulation).

Assessing the risk of coagulation will help to predict thromboembolic complications in the postoperative period and recommend adequate thromboprophylaxis.

Ultrasound investigation will be used to confirm the diagnosis of PAS disorders. The main ultrasound signs of placenta accreta include placental lacunae, disappearance of the normal hypoechoic retroplacental zone, abnormal structure of the border between the uterus and the bladder wall, and a pathological pattern of blood flow with color Doppler mapping. Loss of the hypoechoic retroplacental zone and subplacental hypervascularization is more common in placenta increta, while vascular lacunae and “uterine hernia” are associated with placenta percreta. Ultrasound diagnostics will be carried out using a Philips ultrasound machine, equipped with a 9.4 Hz vaginal probe and a 5.1 Hz convex probe with Doppler.

Magnetic resonance imaging (MRI) of the pelvic organs without contrast is used to confirm the diagnosis and degree of placenta accreta, particularly in cases where the placenta is located on the posterior wall, in suspected placenta percreta, unsatisfactory visualization (in obese women), and to determine angiometric parameters of the abdominal aorta before endovascular intervention. MRI will be carried out using the PHILIPS Ingenia 3.0T device.

Pathohistological examination of the placental and eventual resected uterine tissue will be performed after cesarean section to confirm placenta accreta spectrum syndrome (32, 33). The histological examination will confirm the diagnosis of placenta accreta by extended areas of missing decidua between the placenta and myometrium. These may include an area with placental villi affixed directly to the myometrium or abnormal implantation with a coating of fibrinoid and intervening trophoblast between the placental villi and the myometrium. In cases of placenta accreta, differentiation between decidual cells and extravillous trophoblastic cells can be challenging. The myometrium is typically not thinned in such cases. In contrast to placenta accreta, in cases of placenta increta, the presence of chorionic villi within the myometrium with thinning of the myometrium is expected. For placenta percreta, no remaining myometrium is identified, and dye is present in tissue obtained from areas suspicious for placenta percreta. Invasion through the myometrial wall is observed in the area of percreta or villous tissue adjacent to extrauterine structures (bladder) (32, 33).

### 2.6 Interventions

#### 2.6.1 REBOA

**Figure 2:**
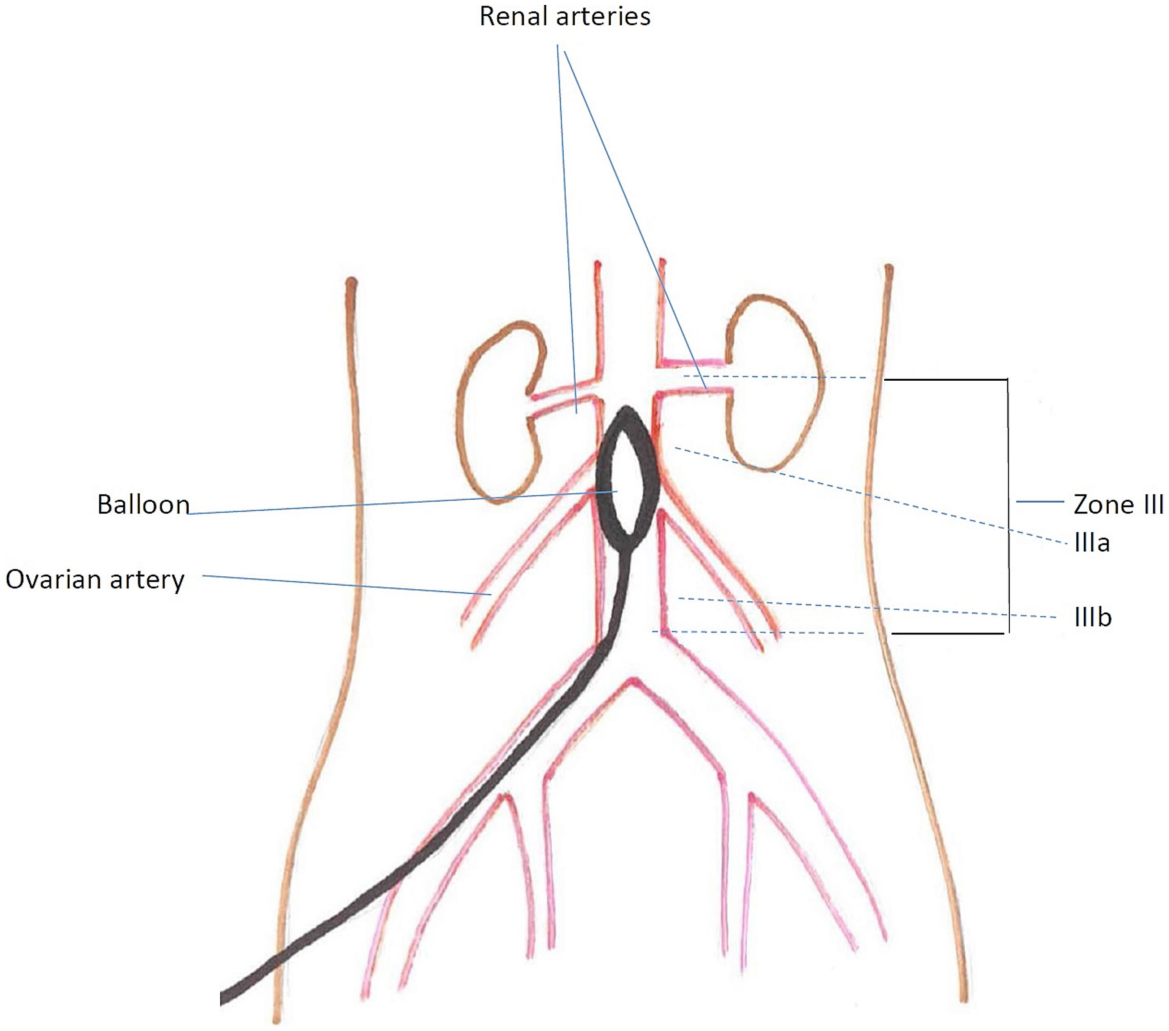
Schema showing REBOA balloon placement in aortic zone IIIa (proximal zone).

In the operating room, a patient will be positioned on the operating table (supine position). After appropriate sanitation of the surgical field, under local anesthesia S. Lidocaine 1% – 10 mL, the right common femoral artery will be punctured under ultrasound guidance. An introducer of the appropriate diameter will be installed and washed with heparinized saline. A compliant or semi-compliant balloon of the appropriate diameter will be installed into the lumen of the aorta Zone IIIa (proximal zone), (Figure 2) under ultrasound guidance. After that, a team of obstetricians will perform a cesarean section and extraction of the fetus. At the time of fetus extraction, the balloon will be inflated in the lumen of the aorta with a saline solution using a 20-mL injection syringe (16, 34–36). Manual control for the absence of pulsation in the common femoral arteries on both sides will be performed for the control group. The maximum inflation time of the balloon will be 15 min. If necessary, the balloon can be inflated repeatedly, with a break of 5 min between periods of aortic occlusion. At the end of the main stage of the operation, the balloon will be deflated step by step under the control of invasive blood pressure measurement. The introducer will be removed using suturing devices and manual compression of the puncture site for 10 min. An aseptic pressure bandage will be applied at the end.

#### 2.6.2 Cesarean section

Surgery will be performed according to the standard procedure following the national guideline on “Cesarean section” used in the NRCMC (37).

In order to avoid operator variability and differences in surgical techniques, the same persons (team of researchers) will be involved in surgical procedures for both groups (cases and controls).

### 2.7 Ethical considerations

All potential study participants will receive an explicit explanation of the study aims, procedures, and potential outcomes. After the detailed explanations written informed consent will be obtained from patients who agree to participate in this study. The study was approved by the Institutional Ethical Board of the University Medical Center on 10.11.2023, protocol #2023/01-028.

### 2.8 Patient withdrawal

Any study participant who no longer agrees to participate in this study can withdraw at any moment without consequences for their subsequent healthcare. These patients will be managed according to the approved national guidelines (25, 37). Data of patients who decided to withdraw from the study will not be analyzed.

### 2.9 Statistical analysis

Statistical analyses will include both descriptive and inferential methods. Baseline characteristics of the study population will be summarized as mean ± standard deviation (SD) or median with interquartile range (IQR) for continuous variables, and as frequencies and percentages for categorical variables. Inferential analyses will compare group differences in major outcomes using independent samples *t*-tests for normally distributed data or Mann–Whitney U-tests for non-normally distributed data. Categorical outcomes will be assessed using Pearson’s χ^2^ test or Fisher’s exact test, as appropriate. For multi-group comparisons, one-way analysis of variance (ANOVA) or Kruskal–Wallis tests will be used, depending on data distribution.

To quantify the effect of REBOA while adjusting for potential confounders, regression models will be used for continuous outcomes, and classification models will analyze categorical outcomes such as hysterectomy or complications. Python will be used for statistical analyses, with Statsmodels (v0.14.4), SciPy (v1.11.3), and Scikit-learn (v1.3.0) libraries for inferential statistics and modeling; NumPy (v2.1.3) and Pandas (v2.1.1) for data handling; and Matplotlib (v3.8.1) and Seaborn (v0.13.2) for data visualization.

## 3 Anticipated results

### 3.1 Outcomes

The *primary outcome* will be blood loss during cesarean section. Blood loss will be measured with reference to the collected blood in the suction flask in the surgical theater and the weighted surgical swabs.

The following will be measured as *secondary outcomes*:

- Directly linked to the intervention’s effectiveness: (1) occurrence of hysterectomy during cesarean section, (2) blood transfusion volume, (3) duration of surgery, (4) balloon application time, (5) stay in an intensive care unit (ICU), (6) complications (thrombotic events, pain, early and delayed surgical complications), and (7) total days of postsurgical hospital stay;
- Indirectly reflect the intervention effectiveness: neonatal outcomes – (8) Apgar score and (9) admission to neonatal ICU.

### 3.2 Study subjects sociodemographic and clinical characteristics

Based on the study participants’ sociodemographic and clinical data, descriptive statistics will be calculated and presented (Table 2).

**Table 2:** Demographic and clinical data.

Variable	Intervention group	Control group	*p*-value
Age (years)			
Gestational age			
Gravidity			
Parity			
Abortions (spontaneous and artificial)			
Prior history of cesarean sections			
Prior history of uterine surgery			

PAS, placenta accreta spectrum.

### 3.3 Analysis of the REBOA procedure outcomes

Specific scheduled parameters (Table 1) will be measured during the study separately for case (cesarean section + REBOA) and control (cesarean section) groups. The results of the study, as well as possible complications, will be analyzed, compared between the groups, and presented in tables (examples of Tables 3, 4).

**Table 3:** Comparison of case and control groups.

Outcome	Intervention group	Control group	P-value
Intraoperative blood loss			
Intraoperative blood transfusion			
Duration of surgery			
Hysterectomy			
Stay in ICU (days)			
Postsurgical hospital stay (days)			
Neonate Apgar score			
Min 1			
Min 5			
Weight			
Admission to ICU			

ICU, intensive care unit; PAS, placenta accrete spectrum; REBOA, resuscitative endovascular balloon occlusion of aorta.

**Table 4:** Complications in patients after cesarean section and REBOA.

Outcome	Intervention group	Control group	*p*-value
Thrombotic events			
Pelvic or lumbosacral pain			
Fever			
Hysterectomy			
Surgical complications			
Early			
Delayed			
Surgical site infection (uterus, abdominal wall)			
Neonatal complications			
Respiratory distress			
Low blood glucose			
Severe infections			
Metabolic disorders			

ICU, Intensive care unit; PAS, placenta accrete spectrum; REBOA, resuscitative endovascular balloon occlusion of aorta.

## 4 Discussion

Bleeding during cesarean section due to PAS disorders remains a big challenge for obstetricians (38–40). The current study will evaluate the effectiveness of REBOA in preventing major hemorrhage during cesarean section as a primary outcome.

The use of REBOA has been described in several studies with benefits in maternal hemodynamic support and reduction of blood loss during uncontrolled bleeding in cases of PAS disorders (15, 17, 41). However, the previous studies on the effectiveness and reliability of endovascular balloon occlusion in PAS disorders were mostly retrospective and characterized by insufficient samples for statistical significance (18, 42, 43). Analysis of existing guidelines for PAS disorders reveals significant variability in quality and applicability, highlighting the urgent need for adapted recommendations specifically designed for low- and middle-income countries (44). In addition, similar studies have not been conducted in Kazakhstan, and therefore there are no specific recommendations for the utilization of REBOA in our patient population. Moreover, since interventional radiology is a relatively new branch of medicine, the issue of using endovascular methods, especially REBOA, to prevent massive obstetric hemorrhage remains open throughout the world.

The possibility of uterine-preserving surgery in women with PAS disorders will be investigated under the application of REBOA. The majority of studies in this area have shown that endovascular management is effective in reducing blood loss during both organ-sparing surgeries and during cesarean sections with hysterectomy. However, definitive conclusions cannot yet be drawn due to conflicting results, which are attributed to the technical features of these procedures, possible complications, and the lack of standardized approaches (9, 11–14). This study will also aim to identify risk groups for thromboembolic complications, which will help to differentiate patients for minimally invasive interventions in obstetric practice in the future.

Future clinical and research implications. The measurable outcome of endovascular management includes the assessment of maternal intra- and postoperative complications. Major complications associated with endovascular balloon occlusion include distal ischemia, reperfusion syndrome, thrombosis, and lower extremity embolization will be analyzed and discussed in comparison with the results from previous studies. The outcomes of this research will facilitate improvements in the local guidelines on the management of placenta previa and PAS disorders. Moreover, the study results may contribute to the development of new guidelines that should prioritize the practical realities faced by developing countries, including limited access to advanced imaging, specialized surgical teams, and blood transfusion resources.

Study strengths and limitations. This will be the first study in Kazakhstani obstetric practice to investigate the efficacy of endovascular balloon occlusion for the management of pregnancy complicated by PAS disorders. The prospective randomized controlled study design will be the major strength of the study, as it will help define the difference between the two approaches (cesarean section vs. cesarean section and REBOA). A limitation of the study, however, will be the small sample size. However, due to the low prevalence of PAS disorders, the complexity of the study, and budget limitations, this sample size has been determined as appropriate based on the sample size calculation. Another limitation will be the lack of blinding for outcome assessors. However, the nature of the investigation and management procedures does not allow for blinding (45). Therefore, this study is planned as an open-label, where patients, clinicians, and care providers are aware of management (cesarean section + REBOA vs. cesarean section) allocations.

## 5 Conclusion

Massive hemorrhage during the cesarean section for pregnancy complicated with PAS disorders remains a huge issue in obstetric care. Endovascular balloon occlusion of the aorta is expected to minimize intraoperative blood loss during cesarean section, decrease the need for transfusion of blood components, and reduce the time of surgical intervention and the length of patient stay in the hospital. Balloon aortic occlusion is expected to reduce the occurrence of cesarean hysterectomy for PAS disorders, ensuring a fertility-sparing approach.
